# Screening and Genome Analysis of Potential Probiotic *Lactiplantibacillus plantarum* with Anti-*Listeria monocytogenes* Activity from Traditional Fermented Foods

**DOI:** 10.3390/microorganisms13092104

**Published:** 2025-09-09

**Authors:** Zhu Qiao, Xing Guo, Zeying Shan, Shijie Luo, Yangyang Mao, Lu Ren, Tao Wang, Yan Ma, Yingying Liu, Junhe Liu

**Affiliations:** 1 School of Biological and Food Processing Engineering, Huanghuai University, Zhumadian 463000, China15111991279@163.com (S.L.); myan9102@163.com (Y.M.);; 2 College of Food Science and Technology, Northwest University, Xi’an 710069, China; 20219044@nwu.edu.cn; 3 Faculty of Food Science and Engineering, Kunming University of Science and Technology, Kunming 650500, China

**Keywords:** probiotic, safety, antimicrobial, food preservation, *Listeria monocytogenes*

## Abstract

This study aimed to isolate *Lactiplantibacillus plantarum* strains with potent anti-*Listeria* activity, desirable probiotic properties, and safety from traditional Chinese fermented foods. Initial screening of 102 lactic acid bacteria (LAB) isolates yielded 43 strains inhibitory to *Listeria monocytogenes*. Eight *L. plantarum* strains exhibiting strong inhibition zones (>15 mm) were selected for probiotic characterization and safety assessment. Among these, strain Z-5 demonstrated remarkable cell surface properties and a favorable safety profile. Further analysis revealed that strain Z-5 demonstrated significant tolerance to simulated gastrointestinal conditions. Whole-genome sequencing revealed a 3.38 Mb chromosome and identified a complete plantaricin biosynthesis gene cluster encoding Pln A, Pln E, and Pln F. The crude bacteriocin extract exhibited high stability and significantly reduced *L. monocytogenes* counts in milk stored at 4 °C and 25 °C in a concentration-dependent manner. Our integrated phenotypic and genomic analyses confirm *L. plantarum* Z-5 as a promising multifunctional probiotic candidate and natural biopreservative for controlling *L. monocytogenes* in foods.

## 1. Introduction

Foodborne diseases pose a significant global health and economic burden. Among the most concerning pathogens is *Listeria monocytogenes*, a remarkably resilient Gram-positive bacterium capable of causing life-threatening listeriosis, with mortality rates reaching 20–30% in vulnerable populations [1]. Its persistence under challenging conditions—including refrigeration, high salinity, and low pH [2]—facilitates contamination across a wide spectrum of foods, notably ready-to-eat products, dairy, meats, and vegetables [3,4]. Consequently, developing effective strategies to combat *L. monocytogenes* in the food chain remains a critical public health imperative.

Current antimicrobial interventions face significant limitations. Physical methods such as thermal processing, plasma treatment, ultraviolet irradiation, and ultrasound often compromise food quality or incur high costs [5], while chemical preservatives raise consumer safety concerns [6,7]. This has driven substantial interest in biological alternatives, particularly probiotics and their bioactive metabolites, valued for their natural origin, safety (GRAS status), and potential health benefits [8,9].

LAB, especially *L. plantarum*, stand out as prime candidates, naturally occurring in fermented vegetables [10], grape must [11], and dairy products [12]. *L. plantarum* not only contributes to flavor and shelf-life extension but also produces a potent arsenal of antimicrobial compounds, including organic acids, hydrogen peroxide, and bacteriocins [13,14,15]. Bacteriocins, ribosomally synthesized antimicrobial peptides, represent highly promising natural preservatives [16]. Many LAB-derived bacteriocins have been identified and employed in food preservation [17,18]. Notably, bacteriocins produced by *L. plantarum* (plantaricins) exhibit strong efficacy against foodborne pathogens. Specific plantaricins (e.g., bio-LP1 [19], GZ1–27 [20], ZJ316 [21]) demonstrate potent activity, even against multidrug-resistant strains and *L. monocytogenes*. Plantaricin LPL-1 exhibits strong inhibitory activity by disrupting cell membrane integrity, increasing membrane permeability, causing leakage of intracellular materials, and inducing proton motive force collapse, ultimately leading to the death of *L. monocytogenes* [22]. The successful application of bacteriocin-producing *L. plantarum* strains, such as ELPL14 in pasteurized milk, further underscores their practical potential for controlling *L. monocytogenes* contamination [23]. Although current applications show promising practical utility, strains combining robust anti-listerial activity, probiotic properties, and comprehensive safety still need to be further explored.

In this study, we aimed to screen *L. plantarum* strains with robust anti-*Listeria* activity, probiotic properties, and safety. (1) LAB were isolated from fermented foods and screened against *L. monocytogenes*, yielding 43 inhibitory strains. Eight *L. plantarum* isolates with superior inhibition zones (>15 mm) were selected for probiotic characterization, including adhesion metrics (auto-aggregation, hydrophobicity), antibiotic susceptibility, and hemolytic activity. (2) The lead strain, *L. plantarum* Z-5, underwent rigorous tolerance profiling (acid, bile, simulated gastrointestinal conditions), bacteriocin production kinetics, and crude bacteriocin stability assays. (3) Whole-genome sequencing and antiSMASH/BAGEL4 analyses elucidated bacteriocin biosynthesis pathways, linking phenotypic activity to genetic determinants. (4) Validate the efficacy of crude bacteriocin against *L. monocytogenes* in milk.

## 2. Materials and Methods

### 2.1. Isolation and Identification of LAB with Antibacterial Activity

LAB were isolated from fermented food samples: three Jiangshui samples from Tianshui, Gansu, China (designated Jiangshui-1, Jiangshui-2, Jiangshui-3), one Chinese cabbage pickle sample (Pickled cabbage-1), and one cowpea pickle sample from Zhumadian, Henan, China, and two Chinese cabbage pickle samples from Luoyang, Henan, China (Pickled cabbage-2 and Pickled cabbage-3). For pickle samples, 5 g of each were homogenized in 10 mL of sterile 0.9% (*w*/*v*) sodium chloride solution by vortexing for 2 min. Serial dilutions (10^−1^ to 10^−4^) were prepared in sterile 0.9% (*w*/*v*) sodium chloride solution. A 100 μL aliquot of each appropriate dilution was spread onto MRS agar (De Man, Rogosa, and Sharpe) and incubated at 37 °C for 48 h. The resulting colonies were further purified on MRS agar plates, followed by screening through microscopic observation and Gram staining. Antibacterial activity was screened against *L. monocytogenes* ATCC 15313 using the dual-layer agar diffusion method [24]. Selected LAB isolates were identified by 16S rRNA gene sequencing. The 16S rRNA gene was amplified by PCR using universal primers 27F (5′-AGAGTTTGATCCTGGCTCAG-3′) and 1492R (5′-GGTTACCTTGTTACGACTT-3′). The PCR process began with an initial denaturation at 95 °C for 5 min, 30 cycles of 95 °C for 30 s, 55 °C for 30 s, 72 °C for 90 s, and final extension at 72 °C for 10 min. The PCR products were sequenced by a biotechnology company (Shanghai, China). The obtained sequences were aligned with the NCBI database (https://www.ncbi.nlm.nih.gov/) for identification. A phylogenetic tree was constructed using MEGA7.0 software and visualized with iTOL (http://itol.embl.de).

### 2.2. Surface Properties of Selected LAB Strains

#### 2.2.1. Auto-Aggregation Analysis

Auto-aggregation of *L. plantarum* was evaluated following the protocol by Huang et al. [25]. Bacterial cells from an overnight MRS broth culture were harvested by centrifugation (5000× *g*, 10 min, 4 °C), washed twice with sterile phosphate-buffered saline (PBS, pH 7.2), and resuspended in PBS to 10^8^ CFU/mL, and the OD_600_ value was recorded as A0. A 4 mL aliquot of the bacterial suspension was vortexed for 10 s and incubated at 37 °C for 2 h. After incubation, the OD_600_ value of the supernatant was measured and recorded as A1. Auto-aggregation was calculated using the formula: Auto-aggregation (%) = (1 − A1/A0) × 100%. The experiment was conducted in triplicate.

#### 2.2.2. Hydrophobicity Determination

Hydrophobicity was measured according to the method of Ilkin Yucel Sengun [26]. Washed cell suspensions were prepared in PBS (pH 7.2) as described for auto-aggregation, and the OD_600_ value was recorded as A0. A 3 mL aliquot of the bacterial suspension was mixed with 1 mL of xylene and vortexed, then incubated at 37 °C for 1 h to allow phase separation. The aqueous phase was carefully removed, and its OD_600_ value was measured as A1. Hydrophobicity was calculated using the formula: Hydrophobicity (%) = (1 − A1/A0) × 100%. The experiment was conducted in triplicate.

### 2.3. Safety Assessment of Selected LAB Strains

#### 2.3.1. Antibiotic Sensitivity Test

The antibiotic sensitivity of the selected strains was determined using the disc diffusion method [23]. Overnight cultures of bacteria (100 μL at 10^8^ CFU/mL) were spread onto MRS agar plates, and antibiotic discs were placed on the surface of the plates. The plates were incubated at 37 °C for 24 h, and inhibition zones were measured. Antibiotics tested included Penicillin (10 U), Cotrimoxazole (25 μg), Ceftriaxone (30 μg), Chloramphenicol (30 μg), Ampicillin (10 μg), Ciprofloxacin (5 μg), Tetracycline (30 μg), and Erythromycin (15 μg). The experiment was performed in triplicate.

#### 2.3.2. Hemolytic Activity

Hemolytic activity was assessed according to the method of Shehata [27]. Selected *L. plantarum* strains were evenly streaked onto blood agar plates containing 5% (*w*/*v*) sheep blood, with *L. monocytogenes* as a positive control. The plates were incubated at 37 °C for 48 h. The presence of β-hemolysis (clear zone around colonies), α-hemolysis (green zone around colonies), and γ-hemolysis (no zone around colonies) was examined.

### 2.4. Probiotic Tolerance Assays of L. plantarum Z-5

#### 2.4.1. Gastric Acid Tolerance

Acid tolerance was assessed following the method of Xu et al. [28]. In brief, overnight cultures of *L. plantarum* Z-5 grown in MRS broth were inoculated into MRS broth adjusted to pH 2.0, 3.0, and 6.4 (control). After incubation at 37 °C for 0, 1, 2, and 3 h, the LAB population was quantified using the plate count method.

#### 2.4.2. Bile Salt Tolerance

Bile salt tolerance was evaluated according to the method of Almeida et al. [29]. The *L. plantarum* Z-5, cultured overnight in MRS broth, was inoculated into MRS broths containing bile salt concentrations of 0.1%, 0.2%, and 0.3% (*w*/*v*), as well as a control without bile salts. The cultures were incubated at 37 °C, and colony counts were performed on agar plates after 0, 2, 4, and 6 h of incubation.

#### 2.4.3. Simulated Gastrointestinal Tolerance

In vitro test of gastrointestinal tolerance was determined according to the method of Cong et al. [30]. *L. plantarum* Z-5 were overnight cultured in MRS broth at 37 °C. The cultures were then centrifuged (10,000× *g*, 5 min), and the pellets were washed three times with PBS (pH 7.2) to adjust the bacterial concentration to 10^8^ to 10^9^ CFU/mL. Suspensions (2% *v*/*v*) were transferred to simulated gastric (containing 10 mg/mL pepsin at pH 3.0) and intestinal fluids (containing 10 mg/mL of trypsin at pH 6.8) and incubated at 37 °C. Samples from the simulated gastric fluid were collected at 0, 0.5, 1, and 2 h and from the simulated intestinal fluid at 0, 2, 4, and 6 h for colony counting.

### 2.5. Growth Curve and Bacteriocin Production of L. plantarum Z-5

Bacteriocin production kinetics were analyzed using a modified method from Jinjin Pei [31]. *L. plantarum* Z-5 was inoculated at 1% (*v*/*v*) into fresh sterile MRS medium and incubated statically at 37 °C for 36 h. During this period, OD_600_, antimicrobial activity, and pH values were measured every 4 h.

### 2.6. Extraction of Bacteriocin and Determination of Minimal Inhibitory Concentration (MIC)

Crude bacteriocin was extracted from cell-free supernatants (CFSs) of *L. plantarum* Z-5 grown under conditions optimized for production. Extraction followed the method of Hongbiao Li [32]. CFS was obtained by centrifugation (10,000× *g*, 20 min, 4 °C) followed by filter sterilization (0.22 μm). Ethyl acetate was added to the CFS at a ratio of 1:1 (*v*/*v*) in a separatory funnel. The mixture was shaken vigorously for 10 min and allowed to stand for 30 min for phase separation. The upper organic phase was collected. The extraction was repeated twice with fresh ethyl acetate. The combined organic phases were concentrated under reduced pressure using a rotary evaporator. The resulting concentrated extract was dissolved in PBS (pH 7.2) and stored at 4 °C. This preparation is referred to as crude bacteriocin extract. Antimicrobial activity was assessed using the agar diffusion method. The minimal inhibitory concentration (MIC) of crude bacteriocin against *L. monocytogenes* was determined according to our previous method [33].

### 2.7. Bacteriocin Stability Analysis

Crude bacteriocin (300 μL) was treated at 60 °C, 80 °C, 100 °C, and 121 °C for 20 min. Untreated bacteriocin served as a control. The pH was adjusted from 2 to 12 using sterile 0.2 M NaOH or 0.2 M HCl, and the samples were incubated for 4 h. Crude bacteriocin without pH adjustment served as control. The samples were treated with 1 mg/mL of pepsin, trypsin, and papain at 37 °C for 4 h. Crude bacteriocin without protease treatment was used as control. Each treatment was performed in triplicate. Residual antimicrobial activity was determined using the agar well diffusion method with *L. monocytogenes* as the indicator strain.

### 2.8. Whole-Genome Sequencing and Bioinformatics Analysis

#### 2.8.1. Illumina Sequencing

DNA extraction and Illumina sequencing Genomic DNA of *L. plantarum* Z-5 was extracted using the genomic DNA extraction kit (Solarbio Science & Technology, Beijing, China) according to the manufacturer’s instructions. The extracted DNA was utilized for Illumina HiSeq × Ten sequencing. Quality control, statistical analysis, and genome evaluation of the raw data were performed. The genome was assembled using SOAP de novo 2.04, and GapCloser (Version 1.12) was employed to fill and optimize gaps in the initial assembly, yielding the final assembly results.

#### 2.8.2. Annotation of Protein-Coding Genes

The coding genes of Z-5 were annotated using various databases, including GO (Gene Ontology), KEGG (Kyoto Encyclopedia of Genes and Genomes), NR (Non-Redundant Protein Database), eggNOG (Evolutionary Genealogy of genes: Non-supervised Orthologous Groups), and SwissProt. These databases provided functional annotations for the genes.

#### 2.8.3. Bacteriocin Gene Analysis

Secondary metabolite gene clusters in the strain were analyzed using antiSMASH (https://antismash.secondarymetabolites.org). Additionally, the BAGEL4 bacterial core peptide database (http://bagel4.molgenrug.nl/index.php) was used to identify the types of bacteriocins produced by *L. plantarum* Z-5.

#### 2.8.4. Safety Gene Annotation

Safety-related genes in the *L. plantarum* Z-5 genome were identified by comparing with the VFDB (Virulence Factor Database). Antibiotic resistance genes were examined using the ARDB (Antibiotic Resistance Gene Database). Transposon genes were investigated in the gene annotation of *L. plantarum* Z-5. Genes associated with toxin production were searched in the annotation table of the *L. plantarum* Z-5 genome.

### 2.9. Evaluation of Crude Bacteriocin Efficacy in Milk Preservation

The potential of crude bacteriocin extract to inhibit *L. monocytogenes* growth in milk was assessed following Zhao et al. [34]. Overnight cultures of L. monocytogenes were added to fresh BHI medium and cultured to the logarithmic phase. The cultures were then centrifuged (4 °C, 5000× *g*, 10 min) to collect the bacterial cells, which were subsequently washed with sterile water. The bacteria were mixed with an equal volume of sterile fresh milk (purchased from the supermarket). Crude bacteriocin was added to achieve final concentrations of 0, 1 × MIC, and 2 × MIC. The samples were thoroughly mixed, sealed, and incubated at 4 °C and 25 °C. The samples were continuously incubated for 4 days, with sampling taken every 24 h. After gradient dilution, appropriate volumes were plated onto BHI agar plates and incubated at 37 °C for 20 h for colony counting.

### 2.10. Data Analysis

Results are expressed as mean ± standard deviation. Statistical comparisons were performed using one-way analysis of variance (ANOVA) followed by Duncan’s test, with analyses conducted using SPSS v20.0. Statistical significance was defined as *p* < 0.05.

## 3. Results

### 3.1. Isolation, Screening, and Identification of Antimicrobial LAB

**Figure 1:**
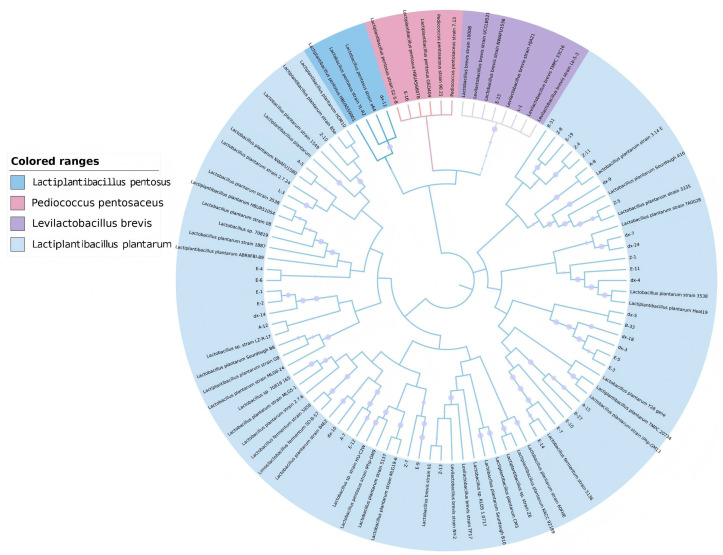
Phylogenetic tree of antibacterial lactic acid bacteria isolated from fermented food according to 16S rDNA sequences.

Initial screening identified 110 bacterial isolates, of which 102 were confirmed as LAB by 16S rRNA sequencing. Among these LAB, 43 strains exhibited inhibitory activity against *L. monocytogenes* (Table 1), with representative species including *Lactiplantibacillus plantarum*, *Levilactobacillus brevis*, *Pediococcus pentosaceus*, and *Lactiplantibacillus pentosus* (phylogenetic distribution in Figure 1). Notably, 20 strains generated inhibition zones exceeding 15 mm in diameter. Eight *L. plantarum* isolates (designated L-4, Z-4, Z-5, Z-15, A-7, A-8, A-12, A-15) demonstrated the most potent antagonistic activity and were selected for further characterization.

### 3.2. Auto-Aggregation

**Figure 2:**
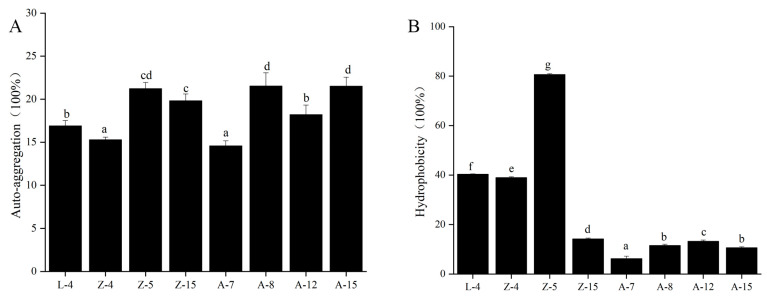
Surface Properties of eight *L. plantarum* strains. (**A**) Auto-aggregation. (**B**) Hydrophobicity. Different lowercase letters (a–g) indicate significant differences among groups (*p* < 0.05).

Surface adhesion properties of the eight selected *L. plantarum* strains were quantified (Figure 2). Auto-aggregation capacity ranged from 15.70% to 23.64%, with strain A-15 exhibiting the highest value (23.64%) (Figure 2A). 

### 3.3. Hydrophobicity

The hydrophobicity of the tested *L. plantarum* strains is depicted in Figure 2B. The hydrophobicity values ranged from 6.20% to 80.62%. Z-5 showed significantly higher hydrophobicity (80.62%) than other strains (*p* < 0.05).

### 3.4. Safety Evaluation

Antibiotic susceptibility profiling indicated universal resistance to ciprofloxacin across all eight strains (Table 2). Strains L-4 and Z-4 showed moderate sensitivity to ceftriaxone, while Z-15 was moderately sensitive to both penicillin and ceftriaxone. Strain A-7 was resistant to sulfamethoxazole and moderately sensitive to erythromycin. Strains Z-5, A-8, A-12, and A-15 were sensitive to the other seven antibiotics tested: penicillin, cotrimoxazole, ceftriaxone, chloramphenicol, ampicillin, tetracycline, and erythromycin.

Hemolytic activity analysis of the eight *L. plantarum* strains revealed no hemolysis on blood agar plates, indicating that these strains exhibit non-hemolytic or γ-hemolytic activity.

### 3.5. Probiotic Function of L. plantarum Z-5

**Figure 3:**
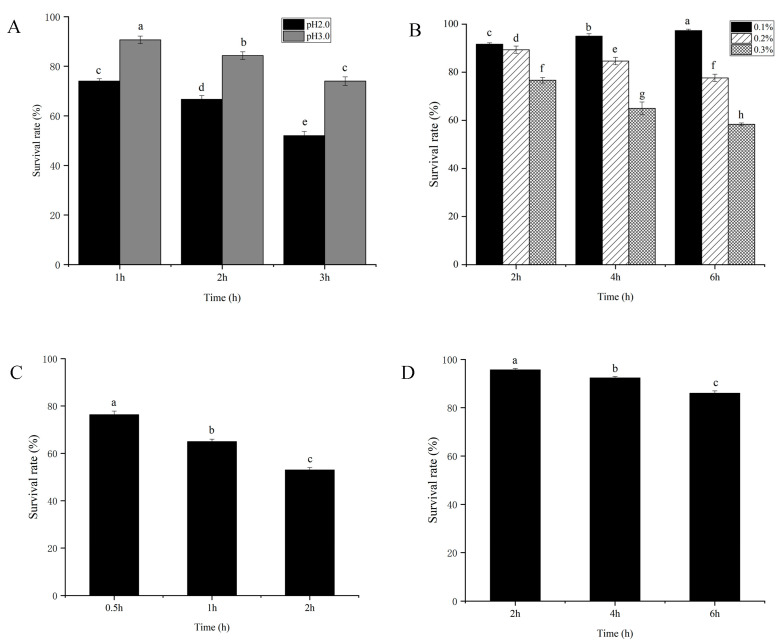
Tolerance analysis of *L. plantarum* Z-5. (**A**) Gastric acid. (**B**) Bile salt. (**C**) Simulated gastric fluid. (**D**) Simulated intestinal fluid. Different lowercase letters (a–h) indicate significant differences among groups (*p* < 0.05).

The assessment of the tolerance of *L. plantarum* Z-5 to pH, bile salts, and gastrointestinal fluids allows for the evaluation of its resistance to the gastrointestinal environment. The acid tolerance of *L. plantarum* Z-5 is shown in Figure 3A. *L. plantarum* Z-5 demonstrated high acid tolerance, maintaining viability at pH 2.0 and 3.0 during a 3 h exposure. Survival rates after 1 h incubation were 74.0% (pH 2.0) and 90.6% (pH 3.0) relative to the initial count (0 h). While viability gradually declined at pH 2.0 to 66.6% and 52.0% after 2 h and 3 h, respectively. The strain exhibited enhanced survival at pH 3.0, reaching 84.3% and 74.1% at the same timepoint. The bile salt tolerance of *L. plantarum* Z-5 is shown in Figure 3B. In environments containing 0.1%, 0.2%, and 0.3% bile salts, after 6 h of incubation, the survival rates are 97.3%, 77.6%, and 58.3%, respectively. The strain exhibited good tolerance to 0.1%, 0.2%, and 0.3% bile salts. The tolerance of *L. plantarum* Z-5 to simulated gastric and intestinal fluids is shown in Figure 3. The survival rates of strain Z-5 in simulated gastric fluid at 0.5, 1, and 2 h were 76.3%, 65.0%, and 53.1%, respectively (Figure 3C). In simulated intestinal fluid, after incubation for 2, 4, and 6 h, the survival counts were 95.6%, 92.3%, and 86.0%, respectively (Figure 3D). These results demonstrate the strain’s good tolerance to simulated gastric and intestinal fluids.

### 3.6. Growth Dynamics and Antibacterial Activity of L. plantarum Z-5

**Figure 4:**
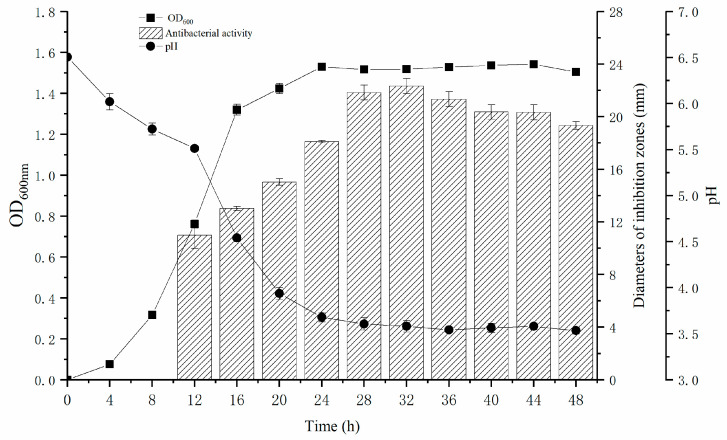
Growth curve, pH variation and inhibitory zone diameter of *L. plantarum* Z-5.

As illustrated in Figure 4, *L. plantarum* Z-5 enters its logarithmic growth phase at 4 h, reaching its maximum biomass at 24 h. The strain remains in a stationary phase between 24 and 44 h, after which it enters the decline phase. Concurrently, the antibacterial activity of the cell-free supernatant, as indicated by the inhibition zone diameter, begins to increase noticeably at 12 h, peaking during the stationary phase (32 h). The fluctuation in inhibition zone diameter reflects the changes in bacteriocin activity, suggesting that bacteriocin secretion commences during the logarithmic phase and reaches its peak during the stationary phase, in alignment with the growth dynamics of Z-5. The pH of the culture medium decreases rapidly during the logarithmic phase, reaching a stable minimum value of 3.6 at 24 h.

### 3.7. Stability of the Crude Bacteriocin

Bacteriocin from strain Z-5 was partially purified by organic solvent extraction. The crude bacteriocin exhibited a MIC of 1.65 mg/mL against *L. monocytogenes*. In the acid and alkali tolerance test (Table 3), the bacteriocin maintained high antimicrobial activity after exposure to extreme pH conditions (pH 2 and pH 12), with maximum activity observed at pH 8, retaining 97.6% of its activity. After treatment at 60 °C and 80 °C for 20 min, the bacteriocin retained 100% of its activity. However, as the temperature increased, the antimicrobial activity gradually decreased, with 88.2% of the activity remaining after treatment at 121 °C for 20 min. The bacteriocin’s activity also showed varying degrees of reduction following treatment with three different proteases, retaining 74.6%, 76.2%, and 85.7% of its activity after treatment with trypsin, pepsin, and papain, respectively, indicating that the bacteriocin possesses proteinaceous properties and good resistance to digestive enzymes.

### 3.8. Genome Assembly and Characteristics

**Figure 5:**
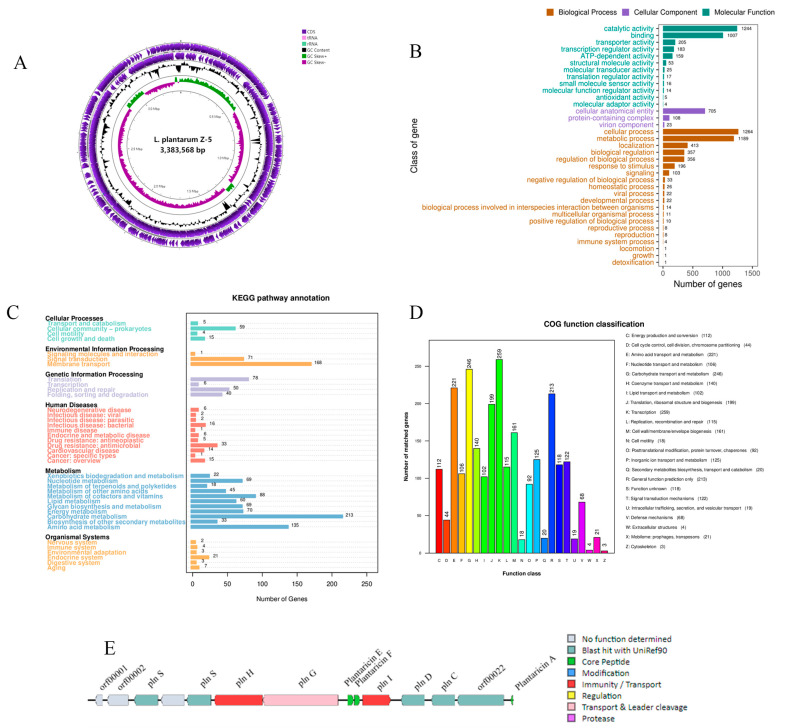
Genome analysis of *L. plantarum* Z-5. (**A**) Circular genome map (from the inside to the outside, the first circle represented the scale; the second circle represented the GC-skew; the third circle represented the GC content; the fourth and fifth circles represented the clusters of orthologous groups of proteins (COG), and protein coding sequences (CDSs) were included in them); (**B**) GO functional classification; (**C**) KEGG functional classification; (**D**) COG functional classification; (**E**) clusters of genes related to the synthesis of bacteriocins.

Whole-genome sequencing of *L. plantarum* Z-5 revealed a chromosome length of 3,383,568 bp with a GC content of 44.38%, comprising 3256 complete coding sequences (Figure 5A). The non-coding RNA elements identified include 67 tRNA genes, 13 rRNA genes, and 2 sRNA genes, as detailed in Table 4.

#### 3.8.1. GO Functional Annotation

The amino acid sequences of *L. plantarum* Z-5 were compared against the GO database, resulting in the annotation of 2192 coding genes. These annotations were classified into three major categories: biological process, cellular component, and molecular function, as depicted in Figure 5B. Within the biological process category, 20 functional classes were identified, with cellular process and metabolic process having the highest gene representation, accounting for 1264 and 1189 genes, respectively. In the cellular component category, 3 functional classes were annotated, including 705 genes associated with cellular anatomical entities, 108 genes with protein-containing complexes, and 23 genes with virion components. In the molecular function category, 12 classes were annotated, with catalytic activity having the highest representation, encompassing 1244 genes. These findings suggest that *L. plantarum* Z-5 may possess specific capabilities related to proliferation and stress tolerance.

#### 3.8.2. KEGG Pathway Annotation

KEGG pathway analysis identified 1718 genes involved in 39 KEGG pathways in *L. plantarum* Z-5 (Figure 5C). The enrichment analysis revealed that the functional genes primarily belong to the categories of metabolism, environmental information processing, cellular processes, genetic information processing, and organismal systems. Within the metabolism category, the largest number of genes was associated with carbohydrate metabolism and amino acid metabolism. The biosynthesis of secondary metabolites and the metabolism of terpenoids and polyketides were annotated to 33 and 18 genes, respectively. Terpenoids and polyketides are important secondary metabolites with significant medicinal value. In the environmental information processing category, most functional genes were related to membrane transport, while in the genetic information processing category, the highest number of genes was associated with translation. In the cellular processes category, functional genes were predominantly located in the cellular community-prokaryotes. These results indicate that *L. plantarum* Z-5 exhibits strong capabilities in energy metabolism and substance transport.

#### 3.8.3. COG Functional Annotation

The protein-coding genes of *L. plantarum* Z-5 were annotated using the COG database, as shown in Figure 5D. A total of 2528 genes were annotated and distributed across 23 COG categories. The category with the most annotated genes was transcription, with 259 genes, representing 10.25% of the total annotated genes. Other categories with a high number of annotated genes included carbohydrate transport and metabolism (246 genes, 9.73%), amino acid transport and metabolism (221 genes, 8.74%), and general function prediction (213 genes, 8.42%). These results indicate that *L. plantarum* Z-5 emphasizes functions related to transcription, carbohydrate, and amino acid transport.

#### 3.8.4. Detection of Antibiotic Resistance and Virulence Genes

Within the genome of *L. plantarum* Z-5, a total of 10 related gene annotations associated with five antibiotics (vancomycin, teicoplanin, lincomycin, fluoroquinolone, and fosfomycin) were identified through the Antibiotic Resistance Gene Database (ARDB), and all showed less than 50% similarity to the corresponding genes in the database, indicating a low similarity to known genes. Additionally, 110 putative virulence factor genes were identified through the Virulence Factor Database (VFDB), with most showing less than 60% similarity to the database entries, suggesting a low similarity to known virulence genes. The genome annotation also revealed genes associated with toxin production, such as those encoding phytotoxin phaseolotoxin and hemolysin.

#### 3.8.5. Bacteriocin Gene Analysis and Mining

The genome of *L. plantarum* Z-5 was analyzed using the antiSMASH database, and potential hotspots for secondary metabolite production were identified using BAGEL4 (Figure 5E). A total of 14 open reading frames (ORFs) were identified, including three core peptides: Plantaricin A (Pln A), Plantaricin F (Pln F), and Plantaricin E (Pln E). The Pln F and Pln E belong to class IIb bacteriocins, while Pln A belongs to class IIc bacteriocins (Table 5). ORF00018 encodes the bacteriocin immunity protein Pln I, a member of the membrane-bound protease CAAX family; ORF00036 encodes a histidine kinase related to bacteriocin production; ORF00020 and ORF00021 encode the response regulators Pln D and Pln C; Lan T encodes a bacteriocin ABC transporter, while PlnG encodes an ATP-binding and permease protein; Hly D encodes a helper factor of the ABC transporter Pln H. ORFs 0003 and 0006 encode Pln S, whose function remains unknown.

### 3.9. The Preservation Effect of Crude Bacteriocin in Milk

**Figure 6:**
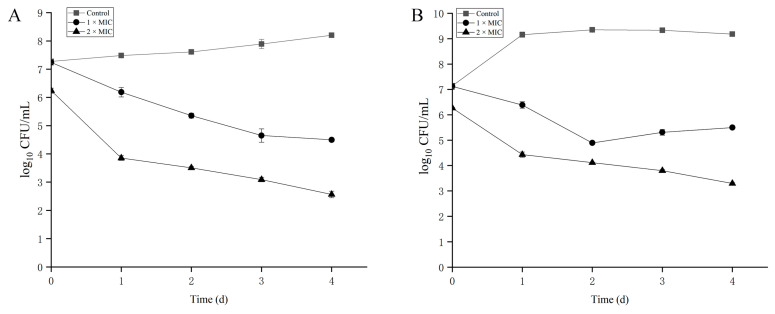
The inhibitory effects of crude bacteriocin produced by *L. plantarum* Z-5 on *L. monocytogenes* in milk at 4 °C (**A**) and 25 °C (**B**).

As shown in Figure 6A,B, *L. monocytogenes* exhibited a growth trend in the control group during the 4-day storage period. Treatment of contaminated milk with bacteriocin at 1× MIC (1.65 mg/mL) significantly reduced viable counts by 2.7 log_10_ CFU/mL at 4 °C and 1.6 log_10_ CFU/mL at 25 °C. Further reductions in viable bacteria were observed during storage following bacteriocin application. At the higher concentration (2× MIC; 3.3 mg/mL), reductions were more pronounced, reaching 3.7 log_10_ CFU/mL at 4 °C and 3.0 log_10_ CFU/mL at 25 °C. Notably, viable counts declined steadily throughout storage at 4 °C post-treatment. In contrast, at 25 °C, bacteriocin treatment induced an initial decrease in viable bacteria followed by a subsequent rebound. These results demonstrate a clear concentration-dependent antibacterial effect of the bacteriocin against *L. monocytogenes*.

## 4. Discussion

The control of foodborne pathogen contamination remains a pressing issue in both higher-income and lower-income countries. Microorganisms show potential in the prevention and control of foodborne pathogens, utilizing antagonistic microorganisms like bacteria, fungi, and yeasts in biological control strategies [35]. Among these, LAB are considered an ideal choice for use as antagonists. Fermented foods provide an optimal habitat for lactic acid bacteria, and selectively isolating strains with specific functional attributes from these foods can enhance both the quality of traditional fermented products and their industrial production. For instance, Martín et al. [3] isolated 371 LAB strains from ready-to-eat cheeses and fermented sausages, identifying 84 with anti-*L. monocytogenes* activity. Similarly, Li et al. [36] screened 200 LAB from fermented dairy samples, identifying 10 strains with strong anti-*Listeria* activity. Fernández et al. [37] demonstrated that 4 LAB could inhibit the growth of *L. monocytogenes* in fermented sausages through bacteriocin production. In this study, 102 LAB strains were isolated from six fermented foods (Table 1), with 43 exhibiting anti-*L. monocytogenes* activity. Eight *L. plantarum* strains showing the strongest inhibition were selected for further analysis.

To further identify the optimal *L. plantarum* strains, we evaluated the probiotic functionality and safety of eight strains. Autoaggregation is a key feature of probiotics, enabling lactic acid bacteria to adhere to epithelial cells and mucosal surfaces, thus facilitating colonization in the gastrointestinal tract [38]. We assessed the autoaggregation capacity of eight *L. plantarum* strains, revealing autoaggregation rates ranging from 15.87% to 23.55%. These results are consistent with those obtained from *L. plantarum* isolated from goat milk [23] and are higher compared to lactic acid bacteria isolated from camel milk (0.5–14.3%) [39]. Autoaggregation represents the initial stage of adhesion, allowing probiotics to form a barrier against pathogen adhesion. Based on these findings, *L. plantarum* Z-5 likely offers a robust barrier effect to inhibit pathogen colonization (Figure 2). In addition to autoaggregation, cell surface hydrophobicity is another important trait of probiotics. Numerous studies have shown that probiotic adhesion correlates positively with both autoaggregation and surface hydrophobicity [26,27]. Hydrophobicity influences the ability of probiotic strains to adhere to and colonize epithelial cells in the human gut, making it a preliminary test for probiotic adhesion [40]. In this study, we measured the hydrophobicity of eight *L. plantarum* strains, with values ranging from 6.2% to 80.62%. Notably, *L. plantarum* Z-5 exhibited a significantly higher hydrophobicity compared to other strains (Figure 2). Das et al. [41] reported hydrophobicity values ranging from 33.4% to 71.57% for five lactic acid bacteria strains. Another study reported a hydrophobicity value of 43.5% for *L. fermentum* 139 [42], all of which are lower than the hydrophobicity of *L. plantarum* Z-5. Thus, the results from both autoaggregation and hydrophobicity tests indicate that *L. plantarum* Z-5 has the potential to prevent pathogen colonization and compete with pathogens through antagonistic interactions.

The safety of probiotics is critical for their subsequent development and utilization. In recent years, the prevalence of antibiotic-resistant microorganisms has increased due to the overuse of antibiotics. When antibiotic-resistant bacteria enter the gastrointestinal tract, they may transfer their antibiotic resistance genes to other bacteria within the gut, posing a potential safety risk to human health [40]. Antibiotic susceptibility testing of eight strains revealed variability in their sensitivity to antibiotics, with all eight strains being sensitive to most antibiotics but resistant to ciprofloxacin. These findings are consistent with studies by Huang [23] and Zhang [43], which reported widespread ciprofloxacin resistance among *L. plantarum* strains. To further assess the safety of these eight strains, we conducted hemolysis tests. Hemolysis is a common virulence factor that can cause anemia and edema in the host. All eight *L. plantarum* strains exhibited γ-hemolysis (no hemolysis) on Columbia sheep blood agar, indicating their safety and potential for application (Table 2).

Based on antimicrobial activity, probiotic functionality, and safety evaluations, *L. plantarum* Z-5 emerged as the most advantageous strain among the eight *L. plantarum*. To further assess *L. plantarum* Z-5, we performed tolerance tests and whole-genome sequencing. Probiotics must possess the ability to endure the harsh conditions of the gastrointestinal tract in order to effectively confer health benefits. The gastrointestinal environment is characterized by factors such as acidity, bile salts, gastric juice, and intestinal fluids, with gastric acidity being the most critical determinant [44]. The pH within the stomach typically ranges from 1.5 to 3.0. In the present study, *L. plantarum* Z-5 exhibited significant acid tolerance. Upon transiting through the biological barriers into the intestinal milieu, probiotics encounter bile salts in the small intestine, typically present at concentrations between 0.03% and 0.3%. Following a 6 h exposure to 0.3% bile salts, *L. plantarum* Z-5 retained a survival rate of 58.3%. Moreover, in simulations of gastric and intestinal conditions, *L. plantarum* Z-5 was able to preserve a high survival rate (Figure 3). Genomic analysis revealed the presence of genes associated with the efflux and regulation of antibiotics such as vancomycin, teicoplanin, lincomycin, fluoroquinolone, and fosfomycin, suggesting potential resistance to these antibiotics. However, no antibiotic synthesis genes were found in the genome. The identification of fluoroquinolone efflux pump genes, consistent with the ciprofloxacin resistance observed in susceptibility testing, may explain the ciprofloxacin resistance of *L. plantarum* Z-5. Further safety assessment of *L. plantarum* Z-5 involved analyzing toxin production-related genes in the genome. Although hemolysin genes were annotated, the absence of hemolytic activity suggests potential gene silencing or non-functional pseudogenes, as observed in *Bacillus coagulans* 13002 [45]. These results confirm that *L. plantarum* Z-5 is safe and holds potential for use in food production.

*L. plantarum* Z-5 exhibits significant inhibitory activity against *L. monocytogenes*. To further analyze the antimicrobial metabolites produced by *L. plantarum* Z-5, we examined its growth and antimicrobial activity dynamics. The results indicated that, during the stationary phase, the fermentation supernatant of *L. plantarum* Z-5 exhibited high antimicrobial activity, suggesting that the antimicrobial substances are likely secondary metabolites. This pattern is similar to the bacteriocin production of *Lactobacillus coryniformis* XN8 [46], indicating that *L. plantarum* Z-5 may inhibit *L. monocytogenes* growth through bacteriocin metabolism. To further analyze these metabolites, we used ethyl acetate extraction to obtain a crude bacteriocin sample, similar to the extraction process used for plantaricin DL3 [47]. Stability tests of the crude bacteriocin sample revealed good thermal and pH stability (Table 3), comparable to bacteriocins such as enterocin TJUQ1 [18] and MXJ 32 [48]. Additionally, the crude bacteriocin sample from *L. plantarum* Z-5 exhibited good enzyme stability, with reduced antimicrobial activity following treatment with three different enzymes, indicating the presence of proteinaceous antimicrobial substances.

To further elucidate the antimicrobial characteristics of *L. plantarum* Z-5, we conducted a whole-genome analysis to identify its bacteriocin gene clusters. Li et al. [32] utilized traditional isolation and purification combined with whole-genome analysis to identify pediocin PA-1. Similarly, Yi et al. [5] used whole-genome analysis to discover two bacteriocins, pentocin DZ1 and pentocin DZ2, in *Lactiplantibacillus pentosus* DZ35. Our analysis of the *L. plantarum* Z-5 genome revealed a bacteriocin gene cluster encoding three bacteriocins: Pln A, Pln F, and Pln E. Of these, Pln F and Pln E are class IIb bacteriocins produced by *L. plantarum*, characterized as dipeptide bacteriocins where antimicrobial activity relies on interactions between the peptides or synergistic effects when present in similar quantities [49]. Pln A is a class IIc bacteriocin, also a peptide bacteriocin inducer [12]. The synthesis and secretion of bacteriocins are believed to require at least four operons: (a) a resistance gene encoding an immunity protein; (b) a structural gene encoding the bacteriocin; (c) a transport gene encoding the ABC transporter; and (d) a regulatory gene encoding the regulatory protein [35]. The *L. plantarum* Z-5 genome contains a complete bacteriocin gene cluster (Figure 5E), including the bacteriocin structural genes (*pln F* and *pln E*), a dedicated immunity protein gene (*pln I*), an ABC transporter gene (*Lan T*), and its accessory proteins (Pln A, Pln D, and Pln C), meeting the requirements for complete bacteriocin synthesis and secretion. Similarly, Zhao et al. [50] found that the genome of *L. plantarum* 163 contains an operon encoding plantaricin PlnEF, while Shu et al. [51] identified multiple bacteriocin genes, including plantaricin PlnEF, in *L. plantarum* SC27, which exhibits broad-spectrum antimicrobial activity. Furthermore, the crude bacteriocin extract of *L. plantarum* Z-5 was applied to milk, demonstrating a strong inhibitory effect against *L. monocytogenes*. In summary, *L. plantarum* Z-5 demonstrates excellent antimicrobial and probiotic properties, making it a potential probiotic strain.

## 5. Conclusions

This study establishes *L. plantarum* Z-5 as a multifunctional probiotic strain with exceptional anti-*Listeria* activity, gastrointestinal resilience, and biosafety. Genomic analysis revealed a complete plantaricin biosynthesis cluster, elucidating the molecular basis for its potent antimicrobial action. The bacteriocin’s stability under thermal, pH, and enzymatic stress, coupled with its efficacy in reducing *L. monocytogenes* in milk, underscores its potential as a natural food preservative. Future work should focus on elucidating the metabolic regulation of bacteriocin biosynthesis, optimizing bacteriocin production and validation in complex food matrices to facilitate industrial translation.

## Figures and Tables

**Table 1:** Antibacterial LAB strains isolated from fermented foods.

No.	Isolate Codes	Indicator	Source	No.	Isolate Codes	Indicator	Source
1	dx-1	-	Jiang shui-1	52	A-4	-	Pickled cowpea
2	dx-2	-	Jiang shui-1	53	A-5	++	Pickled cowpea
3	dx-3	++	Jiang shui-1	54	A-6	-	Pickled cowpea
4	dx-4	+++	Jiang shui-1	55	A-7	+++	Pickled cowpea
5	dx-5	+++	Jiang shui-1	56	A-8	+++	Pickled cowpea
6	dx-6	-	Jiang shui-1	57	A-9	-	Pickled cowpea
7	dx-7	+++	Jiang shui-1	58	A-10	-	Pickled cowpea
8	dx-8	-	Jiang shui-1	59	A-11	-	Pickled cowpea
9	dx-9	-	Jiang shui-1	60	A-12	+++	Pickled cowpea
10	dx-10	+++	Jiang shui-1	61	A-13	-	Pickled cowpea
11	dx-11	+++	Jiang shui-1	62	A-14	-	Pickled cowpea
12	dx-12	-	Jiang shui-1	63	A-15	+++	Pickled cowpea
13	dx-13	-	Jiang shui-1	64	A-16	-	Pickled cowpea
14	dx-14	++	Jiang shui-1	65	A-17	+++	Pickled cowpea
15	dx-15	-	Jiang shui-1	66	A-18	-	Pickled cowpea
16	dx-16	++	Jiang shui-1	67	A-19	-	Pickled cowpea
17	dx-17	-	Jiang shui-1	68	A-20	-	Pickled cowpea
18	dx-18	++	Jiang shui-1	69	A-21	-	Pickled cowpea
19	dx-19	-	Jiang shui-1	70	B-21	-	Jiang shui-3
20	dx-20	-	Jiang shui-1	71	B-22	-	Jiang shui-3
21	dx-21	-	Jiang shui-1	72	B-23	-	Jiang shui-3
22	dx-22	-	Jiang shui-1	73	B-24	-	Jiang shui-3
23	dx-23	-	Jiang shui-1	74	B-25	-	Jiang shui-3
24	dx-24	+++	Jiang shui-1	75	B-27	-	Jiang shui-3
25	ln-1	-	Jiang shui-2	76	B-28	-	Jiang shui-3
26	ln-2	-	Jiang shui-2	77	B-29	-	Jiang shui-3
27	ln-3	-	Jiang shui-2	78	B-30	-	Jiang shui-3
28	ln-4	-	Jiang shui-2	79	B-31	-	Jiang shui-3
29	Z-1	++	Pickled cabbage-1	80	B-32	-	Jiang shui-3
30	Z-2	-	Pickled cabbage-1	81	B-33	-	Jiang shui-3
31	Z-3	-	Pickled cabbage-1	82	B-34	-	Jiang shui-3
32	Z-4	++++	Pickled cabbage-1	83	B-35	-	Jiang shui-3
33	Z-5	++++	Pickled cabbage-1	84	E-1	++	Pickled cabbage-3
34	Z-6	-	Pickled cabbage-1	85	E-2	++	Pickled cabbage-3
35	Z-7	+++	Pickled cabbage-1	86	E-3	++	Pickled cabbage-3
36	Z-8	-	Pickled cabbage-1	87	E-4	+	Pickled cabbage-3
37	Z-9	-	Pickled cabbage-1	88	E-5	++	Pickled cabbage-3
38	Z-10	+++	Pickled cabbage-1	89	E-6	++	Pickled cabbage-3
39	Z-11	+++	Pickled cabbage-1	90	E-7	+	Pickled cabbage-3
40	Z-12	-	Pickled cabbage-1	91	E-9	+	Pickled cabbage-3
41	Z-13	+++	Pickled cabbage-1	92	E-10	++	Pickled cabbage-3
42	Z-14	-	Pickled cabbage-1	93	E-11	++	Pickled cabbage-3
43	Z-15	+++	Pickled cabbage-1	94	E-12	++	Pickled cabbage-3
44	Z-16	-	Pickled cabbage-1	95	E-13	+	Pickled cabbage-3
45	L-1	+++	Pickled cabbage-2	96	E-14	++	Pickled cabbage-3
46	L-2	-	Pickled cabbage-2	97	E-15	-	Pickled cabbage-3
47	L-3	++	Pickled cabbage-2	98	E-16	++	Pickled cabbage-3
48	L-4	++++	Pickled cabbage-2	99	E-18	-	Pickled cabbage-3
49	A-1	-	Pickled cowpea	100	E-19	+	Pickled cabbage-3
50	A-2	-	Pickled cowpea	101	E-20	++	Pickled cabbage-3
51	A-3	-	Pickled cowpea	102	E-21	-	Pickled cabbage-3

+: diameter of inhibition zone 5–10 mm. ++: diameter of inhibition zone 10–15 mm. +++: diameter of inhibition zone 15–20 mm.++++: diameter of inhibition zone >20 mm. -: antimicrobial activity not detected.

**Table 2:** Biosafety properties of LAB strains.

Strains	Antibiotic Resistance								Hemolytic Activity
	P	CIP	CTM	CRO	CHL	AMP	TRC	ERY	
L-4	S	R	S	I	S	S	S	S	γ
Z-4	S	R	S	I	S	S	S	S	γ
Z-5	S	R	S	S	S	S	S	S	γ
Z-15	I	R	S	I	S	S	S	S	γ
A-7	S	R	R	S	S	S	S	I	γ
A-8	S	R	S	S	S	S	S	S	γ
A-12	S	R	S	S	S	S	S	S	γ
A-15	S	R	S	S	S	S	S	S	γ

P, Penicillin; CIP, Ciprofloxacin; CTM, Cotrimoxazole; CRO, Ceftriaxone; CHL, Chloramphenicol; AMP, Ampicillin; TRC, Tetracycline; ERY, Erythromycin; S, Sensitive; I, Intermediate; R, Resistant.

**Table 3:** Stability of the crude bacteriocin produced by *L. plantarum* Z-5.

Treatment		Residual Inhibitory Activity (%)
Heat	60 °C, 20 min	100%
	80 °C, 20 min	100%
	100 °C, 20 min	91.2%
	121 °C, 20 min	88.2%
pH	2	74.4%
	4	76.2%
	6	95.2%
	8	97.6%
	10	84.1%
	12	84.9%
Enzymes	Trypsin	74.6%
	Pepsin	76.2%
	Papain	85.7%

**Table 4:** Genome attributes of *L. plantarum* Z-5.

Attributes	*L. plantarum* Z-5
Genome Size	3,383,568 bp
GC content	44.38%
Genes	3256
tRNA	67
rRNA	13
sRNA	2

**Table 5:** Prediction of *L. plantarum* Z-5 bacteriocins.

Bacteriocin	AA	Molecular Weight (KDa)
Plantaricin A	LQMGATAIKQVKKLFKKWGW	2.36
Plantaricin E	MLQFEKLQYSRLPQKKLAKISGGFNRGGYNFGKSVRHVVDAIGSVAGIRGILKSIR	6.19
Plantaricin F	MKKFLVLRDRELNAISGGVFHAYSARGVRNNYKSAVGPADWVISAVRGFIHG	5.73

## Data Availability

The original contributions presented in this study are included in the article. Further inquiries can be directed to the corresponding authors.

## References

[1] Manville E., Kaya E.C., Yucel U., Boyle D., Trinetta V. (2023). Evaluation of *Listeria monocytogenes* biofilms attachment and formation on different surfaces using a CDC biofilm reactor. Int. J. Food Microbiol..

[2] Park Y.J., Kim Y.J., Yu H.H., Lee N.-K., Paik H.-D. (2023). Cell-free supernatants of *Bacillus subtilis* and *Bacillus polyfermenticus* inhibit *Listeria monocytogenes* biofilm formation. Food Control.

[3] Martín I., Rodríguez A., Alía A., Martínez R., Córdoba J.J. (2022). Selection and characterization of lactic acid bacteria with activity against *Listeria monocytogenes* from traditional RTE ripened foods. LWT.

[4] Jakobsen A.N., Hoel S. (2025). Controlling Listeria monocytogenes in ready-to-eat salmon products using bioprotective cultures of lactic acid bacteria: What hinders the transition from research to industrial application?. Int. J. Food Microbiol..

[5] Yi L., Qi T., Hong Y., Deng L., Zeng K. (2020). Screening of bacteriocin-producing lactic acid bacteria in Chinese homemade pickle and dry-cured meat, and bacteriocin identification by genome sequencing. LWT.

[6] Liu X., Xia X., Liu Y., Li Z., Shi T., Zhang H., Dong Q. (2024). Recent advances on the formation, detection, resistance mechanism, and control technology of *Listeria monocytogenes* biofilm in food industry. Food Res. Int..

[7] Nasrollahzadeh A., Mokhtari S., Khomeiri M., Saris P.E.J. (2022). Antifungal Preservation of Food by Lactic Acid Bacteria. Foods.

[8] Panebianco F., Lovisolo S., Rubiola S., Civera T., Di Ciccio P. (2024). Will *Listeria monocytogenes* biofilm in the food industry withstand the eco-friendly technologies? Recent findings on electrolyzed water, plasma-activated water, ozone, and enzymes. Curr. Opin. Food Sci..

[9] Darko N.K.O., Mills-Robertson F.C. (2025). Probiotic potential and antimicrobial effects of lactic acid bacteria isolated from palm wine against foodborne pathogens in Ghana. Food Chem. Adv..

[10] Toushik S.H., Kim K., Ashrafudoulla M., Mizan M.F.R., Roy P.K., Nahar S., Kim Y., Ha S.-D. (2021). Korean kimchi-derived lactic acid bacteria inhibit foodborne pathogenic biofilm growth on seafood and food processing surface materials. Food Control.

[11] Guo S.-J., Li C.-C., Feng Y.-T., Zhou Y.-R., Liu B., Gao Z.-P., Guo C.-F. (2024). Differences among *Lactiplantibacillus plantarum* strains isolated from different fermented foods in their potential cholesterol-lowering properties. Food Biosci..

[12] Zheng X., Liang Q., Zhao B., Song X., Zhang Y. (2024). Whole genome sequencing and analysis of probiotic characteristics for *Lactiplantibacillus plantarum* EL2 isolated from yak yogurt. LWT.

[13] Keska P., Zielinska D., Karbowiak M., Kruk M., Lisiecka U., Stadnik J. (2025). The potential of cell-free supernatants from *Lacticaseibacillus paracasei* B1 and *Lactiplantibacillus plantarum* O24 as antioxidant and antimicrobial agents. Food Chem..

[14] Fiaz Z., Noor F., Ikram A., Chohan T.A., Aslam M.Z., Arshad N. (2024). Identification of novel antimicrobial compounds in colostrum-associated *Lactiplantibacillus plantarum* ZFS 1 and 2 by integrating in vitro, machine learning and bioinformatics approaches. Food Biosci..

[15] Galvez A., Abriouel H., Lopez R.L., Ben Omar N. (2007). Bacteriocin-based strategies for food biopreservation. Int. J. Food Microbiol..

[16] Srinivash M., Krishnamoorthi R., Mahalingam P.U., Malaikozhundan B., Kaviyadharshini M., Keerthivasan M., Rajkannan P., Samy K.K., Gurushankar K., Chung Y.-K. (2025). Antimicrobial, antioxidant and anticancer properties of bioactive bacteriocins produced by *Lactococcus hircilactis* CH4 and *Lactobacillus delbrueckii* GRIPUMSK isolated from homemade fermented dairy products. Int. Dairy J..

[17] Silva S.P.M., Teixeira J.A., Silva C.C.G. (2023). Recent advances in the use of edible films and coatings with probiotic and bacteriocin-producing lactic acid bacteria. Food Biosci..

[18] Qiao X., Du R., Wang Y., Han Y., Zhou Z. (2020). Purification, characterization and mode of action of enterocin, a novel bacteriocin produced by *Enterococcus faecium* TJUQ1. Int. J. Biol. Macromol..

[19] Ismael M., Qayyum N., Gu Y., Zhezhe Y., Cui Y., Zhang Y., Lu X. (2023). Protective effect of plantaricin bio-LP1 bacteriocin on multidrug-resistance *Escherichia coli* infection by alleviate the inflammation and modulate of gut-microbiota in BALB/c mice model. Int. J. Biol. Macromol..

[20] Du H., Chi H., Yao H., Lu Z., Bie X., Zhang C., Zhao H., Lu F., Chen M. (2022). The antibacterial activity of plantaricin GZ1–27 against MRSA and its bio-preservative effect on chilled pork in combination with chitosan. Int. J. Food Microbiol..

[21] Chen L., Gu Q., Li P., Li Y., Song D., Yang J. (2018). Purification and Characterization of Plantaricin ZJ316, a Novel Bacteriocin against *Listeria monocytogenes* from *Lactobacillus plantarum* ZJ316. J. Food Prot..

[22] Wang Y., Qin Y., Zhang Y., Wu R., Li P. (2019). Antibacterial mechanism of plantaricin LPL-1, a novel class IIa bacteriocin against *Listeria monocytogenes*. Food Control.

[23] Huang X., He Y., Zhong C., Zhao K., Shah N.P., Tao X., Wei H. (2023). Screening of probiotic strains of *Lactiplantibacillus plantarum* from Hu sheep and its ability to inhibit Listeria monocytogenes in pasteurized milk. LWT.

[24] Arrioja-Bretón D., Mani-López E., Palou E., López-Malo A. (2020). Antimicrobial activity and storage stability of cell-free supernatants from lactic acid bacteria and their applications with fresh beef. Food Control.

[25] Huang H., Peng F., Li J., Liu Z., Xie M., Xiong T. (2021). Isolation and characteristics of lactic acid bacteria with antibacterial activity against *Helicobacter pylori*. Food Biosci..

[26] Sengun I.Y., Yalcin H.T., Kilic G., Ozturk B., Peker A.K., Terzi Y., Atlama K. (2024). Identification of lactic acid bacteria found in traditional Shalgam juice using 16S rRNA sequencing and evaluation of their probiotic potential in vitro. Food Biosci..

[27] Shehata M.G., Masry S.H.D., Abd El-Aziz N.M., Ridouane F.L., Mirza S.B., El-Sohaimy S.A. (2024). Probiotic potential of lactic acid bacteria isolated from honeybees stomach: Functional and technological insights. Ann. Agric. Sci..

[28] Xu Y., Xiong T., Zhang L., Du T., Madjirebaye P., Zhao M., Kang X. (2025). Novel lactic acid bacteria with anti-hyperglycaemic properties: In vitro screening and probiotic assessment. Food Biosci..

[29] Almeida J.M.d., Maffei J.T., Gebara C., Minafra C., Toledo-Silva B., Gonçalves M.C., Langoni H., Neto A.T., Souza F.N., Silva N.C.C. (2024). Exploring probiotic potential and antimicrobial properties of lactic acid bacteria from cow’s milk. Appl. Food Res..

[30] Cong S., Zhang X., Ji J., Liu X., Hu N. (2024). Isolation and identification of blueberry-derived lactic acid bacteria and their probiotic, antioxidant, and fermentation properties. Food Biosci..

[31] Pei J., Li X., Han H., Tao Y. (2018). Purification and characterization of plantaricin SLG1, a novel bacteriocin produced by *Lb. plantarum* isolated from yak cheese. Food Control.

[32] Li H., Liu T., Zhang X., Xiong Z., Hong Q., Jia S., Lin Y., Wang L., Zhao Y. (2023). Whole-genome sequencing and bacteriocin purification of *Lactiplantibacillus plantarum* HY41 confirms bactericidal and probiotic potential. Int. Biodeterior. Biodegrad..

[33] Qiao Z., Sun H., Zhou Q., Yi L., Wang X., Shan Y., Yi Y., Liu B., Zhou Y., Lü X. (2020). Characterization and antibacterial action mode of bacteriocin BMP32r and its application as antimicrobial agent for the therapy of multidrug-resistant bacterial infection. Int. J. Biol. Macromol..

[34] Zhao D., Wang Q., Lu F., Bie X., Zhao H., Lu Z., Lu Y. (2022). A novel plantaricin 827 effectively inhibits *Staphylococcus aureus* and extends shelf life of skim milk. LWT.

[35] Qi T., Wang S., Deng L., Yi L., Zeng K. (2021). Controlling pepper soft rot by *Lactobacillus paracasei* WX322 and identification of multiple bacteriocins by complete genome sequencing. Food Control.

[36] Li L., Zhang L., Zhang T., Liu Y., Lü X., Kuipers O.P., Yi Y. (2023). (Meta)genomics -assisted screening of novel antibacterial lactic acid bacteria strains from traditional fermented milk from Western China and their bioprotective effects on cheese. LWT.

[37] Fernández M., Hospital X.F., Caballero N., Jiménez B., Sánchez-Martín V., Morales P., Haza A.I., Hierro E. (2023). Potential of selected bacteriocinogenic lactic acid bacteria to control *Listeria monocytogenes* in nitrite-reduced fermented sausages. Food Control.

[38] Wen Fang Wu Wu J., Redondo-Solano M., Uribe L., WingChing-Jones R., Usaga J., Barboza N. (2021). First characterization of the probiotic potential of lactic acid bacteria isolated from Costa Rican pineapple silages. PeerJ.

[39] Ayyash M., Abushelaibi A., Al-Mahadin S., Enan M., El-Tarabily K., Shah N. (2018). In-vitro investigation into probiotic characterisation of *Streptococcus* and *Enterococcus* isolated from camel milk. LWT.

[40] Peng Y.-Y., Zhong S.-Y., Xu X.-L., Liu D.-M. (2023). Analysis of the safety and probiotic properties of *Bifidobacterium longum* B2-01 by complete genome sequencing combined with corresponding phenotypes. LWT.

[41] Das S., Mishra B.K., Hati S. (2020). Techno-functional characterization of indigenous *Lactobacillus* isolates from the traditional fermented foods of Meghalaya, India. Curr. Res. Food Sci..

[42] de Albuquerque T.M.R., Garcia E.F., de Oliveira Araujo A., Magnani M., Saarela M., de Souza E.L. (2018). In Vitro Characterization of *Lactobacillus* Strains Isolated from Fruit Processing By-Products as Potential Probiotics. Probiotics Antimicrob. Proteins.

[43] Zhang B., Wang Y., Tan Z., Li Z., Jiao Z., Huang Q. (2016). Screening of Probiotic Activities of *Lactobacilli* Strains Isolated from Traditional Tibetan Qula, a Raw Yak Milk Cheese. Asian-Australas. J. Anim. Sci..

[44] Vijayalakshmi S., Adeyemi D.E., Choi I.Y., Sultan G., Madar I.H., Park M.-K. (2020). Comprehensive in silico analysis of lactic acid bacteria for the selection of desirable probiotics. LWT.

[45] Wu Y.-p., Liu D.-m., Zhao S., Huang Y.-y., Yu J.-j., Zhou Q.-y. (2022). Assessing the safety and probiotic characteristics of *Bacillus coagulans* 13002 based on complete genome and phenotype analysis. LWT.

[46] Yi L., Dang J., Zhang L., Wu Y., Liu B., Lü X. (2016). Purification, characterization and bactericidal mechanism of a broad spectrum bacteriocin with antimicrobial activity against multidrug-resistant strains produced by *Lactobacillus coryniformis* XN8. Food Control.

[47] Lv X., Lin Y., Jie Y., Sun M., Zhang B., Bai F., Zhao H., Li J. (2017). Purification, characterization, and action mechanism of plantaricin DL3, a novel bacteriocin against *Pseudomonas aeruginosa* produced by *Lactobacillus plantarum* DL3 from Chinese Suan-Tsai. Eur. Food Res. Technol..

[48] Lü X., Yi L., Dang J., Dang Y., Liu B. (2014). Purification of novel bacteriocin produced by *Lactobacillus coryniformis* MXJ 32 for inhibiting bacterial foodborne pathogens including antibiotic-resistant microorganisms. Food Control.

[49] Tang X., Wu S., Wang X., Gu Q., Li P. (2018). Antimicrobial activity and preliminary mode of action of PlnEF expressed in *Escherichia coli* against *Staphylococci*. Protein Expr. Purif..

[50] Zhao D., Meng F., Zhou L., Lu F., Bie X., Sun J., Lu Z., Lu Y. (2021). Maltose effective improving production and regulatory biosynthesis of plantaricin EF in *Lactobacillus plantarum* 163. Appl. Microbiol. Biotechnol..

[51] Shu H., He X., Hong Z., Dong K., Zou Y., Cao M., Wang R., Xu Y., Liao L., Zuo H. (2024). Screening and genome analysis of potential probiotic lactic acid bacteria with broad-spectrum antibacterial activity from Sichuan sun-dried vinegar grains (Cupei). LWT.

